# Propranolol Promotes Glucose Dependence and Synergizes with Dichloroacetate for Anti-Cancer Activity in HNSCC

**DOI:** 10.3390/cancers10120476

**Published:** 2018-11-30

**Authors:** Christopher T. Lucido, W. Keith Miskimins, Paola D. Vermeer

**Affiliations:** Cancer Biology and Immunotherapies Group, Sanford Research, 2301 East 60th St North, Sioux Falls, SD 57104, USA; Chris.Lucido@coyotes.usd.edu (C.T.L.); Keith.Miskimins@sanfordhealth.org (W.K.M.)

**Keywords:** head and neck cancer, head and neck squamous cell carcinoma, human papillomavirus, tumor metabolism, propranolol, DCA, combination therapy

## Abstract

Tumor cell metabolism differs from that of normal cells, conferring tumors with metabolic advantages but affording opportunities for therapeutic intervention. Accordingly, metabolism-targeting therapies have shown promise. However, drugs targeting singular metabolic pathways display limited efficacy, in part due to the tumor’s ability to compensate by using other metabolic pathways to meet energy and growth demands. Thus, it is critical to identify novel combinations of metabolism-targeting drugs to improve therapeutic efficacy in the face of compensatory cellular response mechanisms. Our lab has previously identified that the anti-cancer activity of propranolol, a non-selective beta-blocker, is associated with inhibition of mitochondrial metabolism in head and neck squamous cell carcinoma (HNSCC). In response to propranolol, however, HNSCC exhibits heightened glycolytic activity, which may limit the effectiveness of propranolol as a single agent. Thus, we hypothesized that propranolol’s metabolic effects promote a state of enhanced glucose dependence, and that propranolol together with glycolytic inhibition would provide a highly effective therapeutic combination in HNSCC. Here, we show that glucose deprivation synergizes with propranolol for anti-cancer activity, and that the rational combination of propranolol and dichloroacetate (DCA), a clinically available glycolytic inhibitor, dramatically attenuates tumor cell metabolism and mTOR signaling, inhibits proliferation and colony formation, and induces apoptosis. This therapeutic combination displays efficacy in both human papillomavirus-positive (HPV(+)) and HPV(−) HNSCC cell lines, as well as a recurrent/metastatic model, while leaving normal tonsil epithelial cells relatively unaffected. Importantly, the combination significantly delays tumor growth in vivo with no evidence of toxicity. Additionally, the combination of propranolol and DCA enhances the effects of chemoradiation and sensitizes resistant cells to cisplatin and radiation. This novel therapeutic combination represents a promising treatment strategy which may overcome some of the limitations of targeting individual metabolic pathways in cancer.

## 1. Introduction

Head and neck cancer comprises a histologically diverse group of tumors arising in various anatomic sites within the head and neck, including the sinuses, oral cavity, pharynx, and larynx [1]. Taken as a whole, this group of tumors represents the sixth most common cancer worldwide, with an estimated 650,000 diagnoses and 350,000 deaths annually [1]. Histologically, an overwhelming majority of head and neck tumors belong to the squamous cell carcinoma subtype (head and neck squamous cell carcinoma, HNSCC) [1]. Annually, HNSCC accounts for over 500,000 diagnoses globally [2], with over 50,000 diagnoses and over 10,000 deaths in 2017 in the United States alone [3]. Primary risk factors for HNSCC development include tobacco and alcohol use, which are implicated in approximately 75% of cases, and human papillomavirus (HPV) infection, which is implicated in approximately 25% of cases (HPV(+) HNSCC) [1].

Current standard treatment of HNSCC includes disease stage-based combinations of surgery, radiation, and/or chemotherapy [4]. HPV status holds prognostic value in HNSCC, as HPV positivity is associated with improved treatment response and survival [5]. This dichotomy has lead to heightened interest in recognizing HPV(+) and HPV(−) HNSCC as two distinct diseases. Current research focus areas include the investigation of new therapeutic strategies to improve outcomes, especially in HPV(−) HNSCC patients, and to allow for de-intensification of standard therapies in certain cases of HPV(+) HNSCC to alleviate morbidities associated with overtreatment [4]. Additionally, while management of primary HNSCC is relatively successful regardless of HPV status, recurrent and/or metastatic (R/M) disease is almost universally non-responsive to curative therapy and therapeutic approaches in the R/M setting are associated with significant treatment-associated morbidity [6,7]. Thus, novel therapeutic strategies are required for HNSCC. Ideal additions to current treatment strategies in HNSCC would include therapies that: (1) Are effective in both HPV(+) and HPV(−) HNSCC, (2) are effective in the R/M setting, (3) enhance the effects of currently utilized modalities, allowing for de-intensification of therapy in certain cases, and (4) are well-tolerated. 

Propranolol, a non-selective beta-blocker, has garnered increasing interest for its anti-cancer potential in a number of tumor types, including colorectal, breast, ovarian, pancreatic, and head and neck [8,9,10,11,12,13,14,15]. Retrospective clinical analyses have documented improved outcomes in breast and ovarian cancer patients taking non-selective beta-blockers during the course of their disease [16,17]. Moreover, a recent clinical trial showed that adjuvant propranolol significantly improved progression-free survival in patients with thick cutaneous melanoma [18]. Recently, our laboratory showed that propranolol displays significant anti-cancer effects in a murine model of R/M HPV(+) HNSCC and that these effects are associated with a significant reduction in tumor cell mitochondrial metabolism [19]. While targeting tumor cell metabolism has shown great promise, tumors are metabolically plastic [20]. In the face of single metabolic pathway inhibition, tumor cells are capable of compensating by utilizing other pathways to meet growth and energy demands, thereby limiting the efficacy of some metabolism-targeting drugs [21,22,23]. Along these lines, we show an increase in glycolytic activity concurrent with propranolol-mediated mitochondrial inhibition, indicative of a metabolic compensatory mechanism that may limit the efficacy of propranolol as a single agent. Thus, we hypothesized that propranolol’s metabolic effects would promote a state of enhanced glucose dependence, and that propranolol together with glycolytic inhibition would prove a highly effective therapeutic combination in HNSCC. Here, we show that propranolol synergizes with glucose deprivation for improved anti-cancer activity, and that the rational combination of propranolol and dichloroacetate (DCA), a clinically available glycolytic inhibitor, dramatically attenuates tumor cell metabolism and mTOR signaling, inhibits proliferation and colony formation, and induces apoptosis. This therapeutic combination displays efficacy in both HPV(+) and HPV(−) HNSCC cell lines, as well as an R/M model, while leaving normal tonsil epithelial cells relatively unaffected. Importantly, the combination significantly delays tumor growth in vivo with no evidence of toxicity. Additionally, the combination of propranolol and DCA enhances the effects of and sensitizes resistant cells to chemoradiation. This novel combination of clinically available, metabolism-targeting agents represents an intriguing treatment strategy in HNSCC that may overcome some of the pitfalls associated with targeting single metabolic pathways in cancer.

## 2. Results

### 2.1. Propranolol Alters Tumor Cell Metabolism.

**Figure 1:**
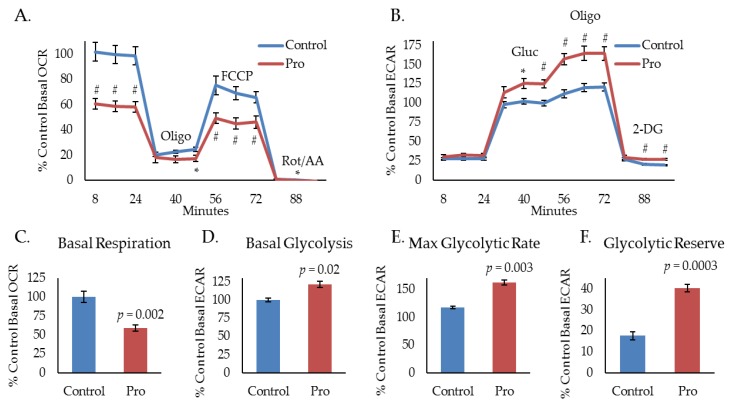
Propranolol alters tumor cell metabolism. Mitochondrial (**A**) and glycolytic stress test (**B**) results of MLM3 cells treated with propranolol (40 μM) showing propranolol-mediated changes in basal mitochondrial respiration (**C**), basal glycolysis (**D**), maximum glycolytic rate (**E**), and glycolytic reserve (**F**). Data are represented as % control basal oxygen consumption rate (OCR) (average of first three measurements) or extracellular acidification rate (ECAR) (average of three measurements immediately following glucose injection). Each data point represents mean +/− SEM of *n* = 9 (mitochondrial stress test) or 5 (glycolytic stress test) independent biological replicates; *p*-values reflect *t*-test results (* *p* ≤ 0.03; # *p* ≤ 0.008).

Our laboratory uses several murine models to study HPV(+) HNSCC: A primary tumor model derived from mouse oropharyngeal epithelial cells stably expressing the HPV oncogenes, E6 and E7, H-Ras, and luciferase (mEERL) [24,25,26,27], and an aggressive, treatment resistant, R/M model derived from a spontaneously arising lung metastasis in a mouse that was implanted with a mEERL tumor and failed chemoradiation treatment (MLM3) [28]. Importantly, these models faithfully recapitulate the human disease [24,25,26,27,28]. Recently, we have shown that propranolol, a non-selective beta-blocker, exhibits significant anti-cancer activity in the aggressive MLM3 model and that its effects are associated with a decrease in oxygen consumption rate (OCR), indicating inhibition of mitochondrial metabolism [19]. To investigate propranolol’s effects on glycolytic activity, we used a Seahorse extracellular flux analyzer to measure extracellular acidification rate (ECAR) during a glycolytic stress test. The glycolytic stress test utilizes sequential injections of glucose (Gluc), oligomycin (Oligo), and 2-deoxyglucose (2-DG) to acquire measurements of basal glycolysis (ECAR following Gluc injection), maximal glycolysis (ECAR following Oligo injection), glycolytic reserve capacity (difference between basal and maximal ECAR), and non-glycolytic acidification (ECAR following 2-DG injection). Interestingly, while mitochondrial stress testing indicates significant inhibition of MLM3 mitochondrial metabolism during treatment with propranolol (Figure 1A,C), glycolytic stress testing shows evidence of heightened glycolytic activity (Figure 1B). In response to propranolol, basal (Figure 1D) and maximal (Figure 1E) glycolytic rates significantly increased, as did glycolytic reserve capacity (Figure 1F). These data indicate a potential compensatory response to propranolol-mediated metabolic inhibition which may limit or mitigate its anti-cancer efficacy as a single agent. 

### 2.2. Propranolol Enhances Glucose Dependence and Synergizes with Glucose Deprivation for Improved Anti-Cancer Activity.

**Figure 2:**
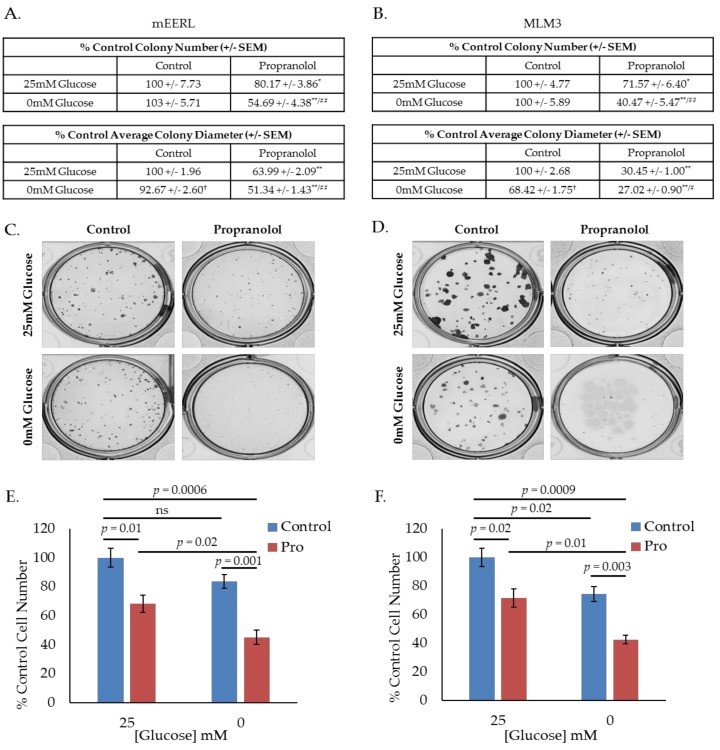
Propranolol enhances glucose dependence and synergizes with glucose deprivation for improved anti-cancer activity. Colony-forming assay results and representative images of mEERL (**A**,**C**) and MLM3 cells (**B**,**D**) treated with propranolol (40 µM) in the presence or absence of glucose (25 mM). Values reflect % colony number (top) or % average colony diameter (bottom) relative to 25 mM glucose control. Each value represents mean +/- SEM of *n* = 6 (mEERL) or 4 (MLM3) independent biological replicates; *p*-values reflect *t*-test results (* *p* ≤ 0.05 and ** *p* ≤ 0.001 comparing control vs. propranolol-treated cells at the same glucose concentration; ^†^ *p* ≤ 0.001 comparing control cells at 25 mM vs. 0 mM glucose; ^#^ *p* ≤ 0.05 and ^##^ *p* ≤ 0.05 comparing propranolol-treated cells at 25 mM vs. 0 mM glucose). Proliferation assay results of mEERL (**E**) and MLM3 cells (**F**) treated with propranolol (40 µM) for 48 h in the presence or absence of glucose. Values reflect % cell number relative to 25 mM glucose control. Each data point represents mean +/− SEM of *n* = 4 independent biological replicates; *p*-values reflect *t*-test results.

Given these results, we hypothesized that propranolol would promote a state of increased glucose dependence and that starving cells of glucose during treatment with propranolol would enhance its effects. To investigate this hypothesis, we conducted colony forming assays using mEERL or MLM3 cells treated with propranolol in the presence or absence of glucose, where colony number and diameter are surrogate measures for survival and proliferation, respectively. Interestingly, though average colony diameter decreased, glucose starvation alone did not significantly alter colony number in either cell line (Figure 2A–D), suggesting that while glucose may be required to promote maximal proliferation, mEERL and MLM3 cells do not require glucose for survival. However, while propranolol alone significantly decreased colony number and diameter in both cell lines, glucose deprivation significantly enhanced these effects (Figure 2A–D). These results are supported by standard cell counting experiments following 48-h treatment with propranolol in the presence or absence of glucose (Figure 2E,F). In all, these data indicate that propranolol promotes glucose dependence, and that propranolol’s anti-cancer activity is augmented by glucose starvation.

### 2.3. Propranolol Synergizes with the Glycolytic Inhibitor DCA to Dramatically Attenuate Tumor Cell Metabolism and mTOR Signaling. 

**Figure 3:**
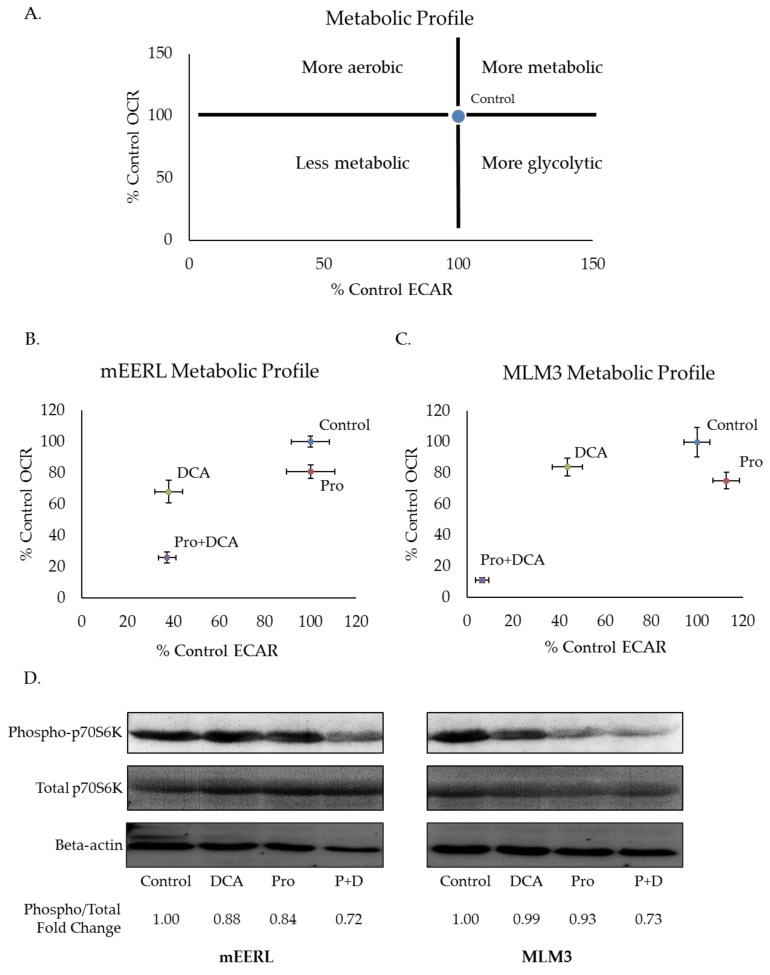
Propranolol synergizes with the glycolytic inhibitor dichloroacetate (DCA) to dramatically attenuate tumor cell metabolism and mTOR signaling. Metabolic profile schematic (**A**), representing possible shifts in metabolic phenotype compared to control. Metabolic profiles of mEERL (**B**) and MLM3 cells (**C**) treated overnight with propranolol (40 µM) and/or DCA (10 mM). Values reflect the average of *n* = 3–5 independent biological replicates +/− SEM. Values were adjusted for non-glycolytic acidification and normalized to total protein. Western blot (**D**) assessing mTOR activity via p70S6K phosphorylation in mEERL (left) and MLM3 cells (right) following overnight treatment with propranolol (40 µM) and/or DCA (10 mM). Spot densitometry was used to calculate fold change in phosphorylated/total p70S6K signal intensity relative to control for each cell line.

Blood glucose levels are tightly regulated. Decreased glucose consumption, as with prolonged fasting, induces glycogenolysis and gluconeogenesis to maintain blood glucose levels and preserve healthy organismal function [29]. As such, completely depriving tumors of glucose is not likely to be a feasible therapeutic strategy. Thus, we sought to investigate propranolol’s effectiveness alongside pharmacologic glycolytic inhibition and focused our efforts on another clinically available agent, dichloroacetate (DCA). DCA is a small molecule with high bioavailability when given orally [30]. Its effects on glycolysis derive from its ability to inactivate pyruvate dehydrogenase kinase (PDK), the negative regulator of pyruvate dehydrogenase (PDH) [30,31]. Releasing PDH from the inhibitory activity of PDK promotes the conversion of pyruvate to acetyl-CoA for subsequent entry into mitochondrial metabolism [30,31]. Thus, DCA limits the pool of pyruvate available for conversion to lactate, and, in doing so, limits the regeneration of NAD^+^ which is required for high rates of glycolytic flux. To investigate the metabolic effects of combining propranolol and DCA, we measured ECAR and OCR via Seahorse extracellular flux analysis. Plotting ECAR and OCR as X and Y coordinates, respectively, allows for visualization of shifts in metabolic phenotypes (Figure 3A). A shift upward and rightward or downward and leftward from control indicates a more or less metabolically active phenotype, respectively. Additionally, a shift rightward and downward indicates a more glycolytic phenotype, whereas a shift leftward and upward indicates a more aerobic phenotype. In both mEERL and MLM3 cells, the combination of propranolol and DCA induced a dramatic shift toward a less metabolically active phenotype that was greater than the sum of their individual effects (Figure 3B,C). This metabolic shift was associated with attenuated mTOR signaling, as assessed by the phosphorylation status of the downstream effector molecule p70S6K (Figure 3D). Our laboratory has previously shown that the mTOR signaling pathway, a master regulatory pathway controlling cellular metabolism and growth, is critical in the progression of both of these tumor models [32,33]. Of note, while mTOR regulates aspects of cellular metabolism, its activity is, in turn, regulated by metabolic cues [34]—thus, whether the apparent decrease in mTOR signaling induced by the drug combination is driving the metabolic shift or vice versa remains to be determined. Nonetheless, these data are indicative of significant metabolic perturbations induced by the combination of propranolol and DCA. 

### 2.4. The Combination of Propranolol and DCA Synergizes for Significant Anti-Cancer Activity in HNSCC, But Has Little Effect on Primary Tonsil Epithelial Cells.

**Figure 4:**
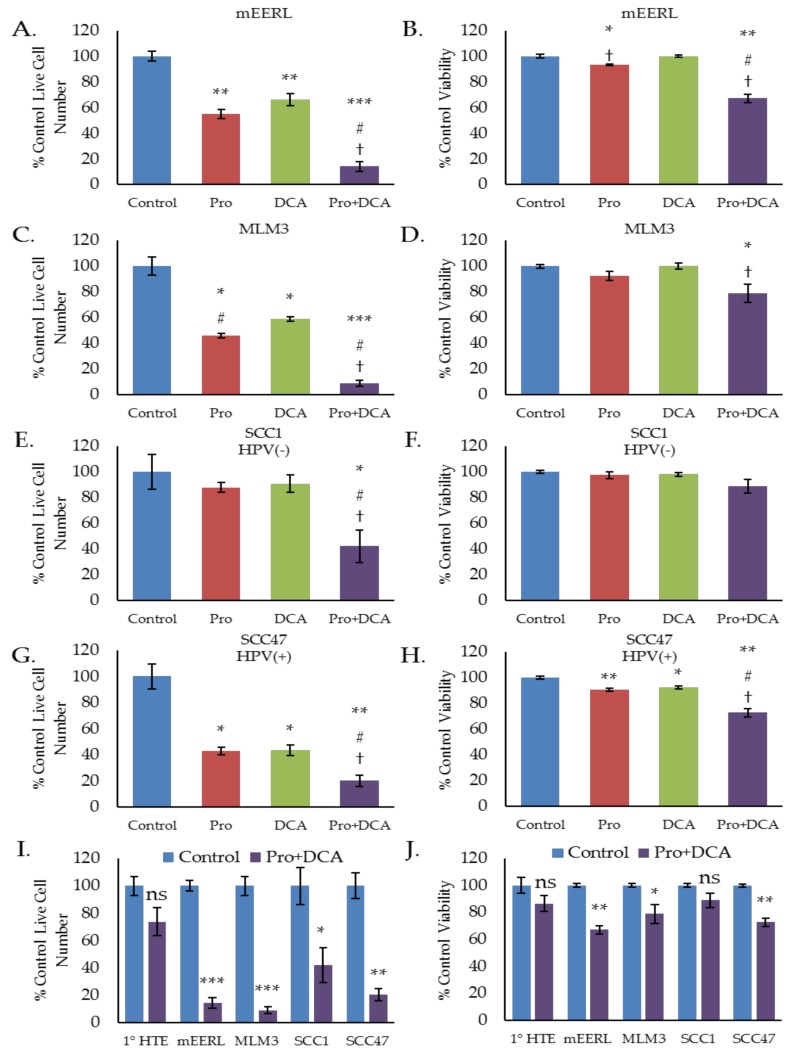
The combination of propranolol and DCA synergizes for robust anti-cancer activity in head and neck squamous cell carcinoma (HNSCC), but has little effect on primary tonsil epithelial cells. Cell counting (**A**,**C**,**E**,**G**,**I**) and trypan blue exclusion results (**B**,**D**,**F**,**H**,**K**) of mEERL (**A,B**), MLM3 (**C,D**), SCC1 (E,F), and SCC47 (**G,H**) treated with propranolol (40 µM) and/or DCA (10 mM) for 48–72 h. SCC1 and SCC47 are human papillomavirus (HPV)(−) and HPV(+) HNSCC cell lines, respectively. Comparison of Pro + DCA effects in primary human tonsil epithelial cells (1° HTE) versus HNSCC cell lines is shown in (**I**) and (**J**). Values reflects % live cell number or % viability relative to control. Each data point represents mean +/− SEM of *n* = 3–4 independent biological replicates; *p*-values reflect *t*-test results (* *p* ≤ 0.05, ** *p* ≤ 0.006, *** *p* ≤ 0.0001 vs. control for a given cell line; # *p* ≤ 0.05 vs. propranolol only; † *p* ≤ 0.05 vs. DCA only; ns = not significant).

**Figure 5:**
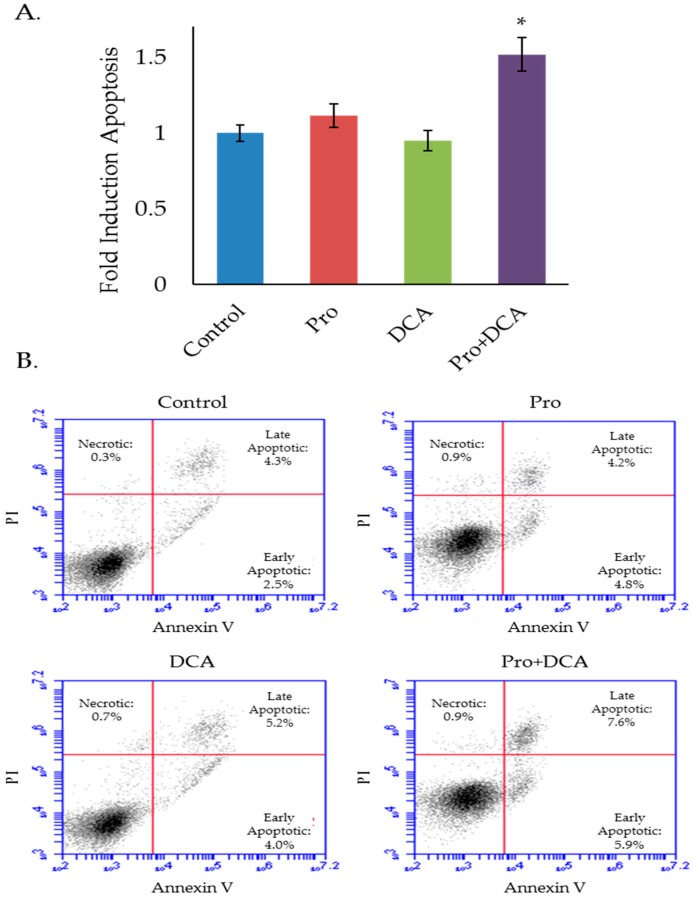
The combination of propranolol and DCA induces apoptosis. Results (**A**) and representative flow cytometry scatter plots (**B**) of MLM3 cells treated with propranolol (40 µM) and/or DCA (10 mM) for 48 h and stained with annexin V and propidium iodide (PI) to assess cell death and apoptosis. Values in (A) reflect fold induction of apoptosis (indicated by positive annexin staining) relative to control. Data were pooled from two independent experiments. Each data point represents mean +/− SEM of *n* = 5 independent biological replicates; *p*-values reflect *t*-test results (* *p* < 0.02 vs. all other treatment groups).

To investigate whether these metabolic effects translate to any differences in cellular proliferation or viability we conducted a series of cell counting and trypan blue exclusion assays, treating cells with propranolol, DCA, or the combination. In both the mEERL and MLM3 cell lines, the combination of propranolol and DCA significantly decreased cell number over either drug alone (Figure 4A,C). The combination was also found to be cytotoxic in both cell lines (Figure 4B,D), where propranolol alone induced only a marginal (but statistically significant) decrease in cell viability in the mEERLs (Figure 4B), and neither drug alone was found to be significantly cytotoxic in the MLM3s (Figure 4D). Cytotoxicity was confirmed via annexin V/propidium iodide staining, which showed that the combination induces apoptosis (Figure 5A,B).

We further investigated these effects using human HNSCC cell lines, SCC1 and SCC47, which are HPV(−) and HPV(+) cell lines, respectively. Similar to the results seen in the mEERL and MLM3 cell lines, the combination of propranolol and DCA significantly decreased both SCC1 and SCC47 cell number as compared to either drug alone (Figure 4E,G). Highlighting this complementary activity, neither drug alone significantly decreased SCC1 cell number, whereas the combination decreased cell number to below 50% of control (Figure 4E). Additionally, while propranolol and DCA marginally (but significantly) reduced cell viability in the SCC47s, the combination significantly augmented cytotoxicity (Figure 4H). The SCC1 cells also displayed a decrease in viability in response to the combination, though this result was not statistically significant (Figure 4F). 

To determine the selectivity of these effects for cancer cells, we treated primary human tonsil epithelial (1° HTE) cells with the combination and compared their response to the responses observed in the cancer cell lines. Neither 1° HTE cell number nor viability was significantly decreased in response to the combination of propranolol and DCA (Figure 4I,J), suggesting that the combination is relatively selective for tumor cells. 

### 2.5. The Combination of Propranolol and DCA Delays Tumor Growth In Vivo

**Figure 6:**
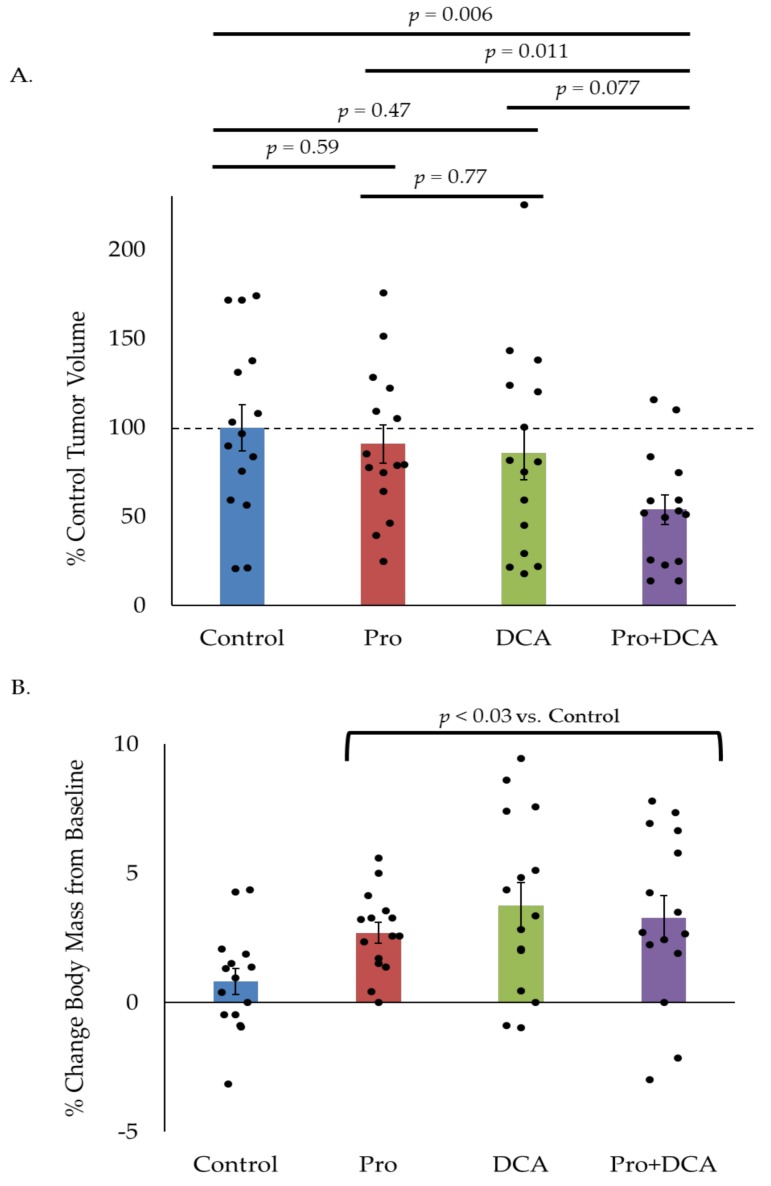
The combination of propranolol and DCA delays tumor growth in vivo. Average tumor volume (**A**) and percent change in body mass (**B**) in mice implanted with MLM3 tumors and treated daily with propranolol, DCA, or the combination. Values in (A) reflect percent tumor volume relative to control animals. Values in (B) reflect percent change in body mass relative to pre-tumor baseline. Each bar represents mean +/− SEM of *n* = 15 mice. Scatter points represent individual animals. *p*-values reflect *t*-test results.

As in vitro results do not always translate to in vivo efficacy, we sought to test the effects of propranolol and DCA on in vivo tumor growth. Mice were implanted in their hind limbs with MLM3 tumors and treated daily with intraperitoneal propranolol (10 mg/kg/day) and/or DCA (6 mg/day) beginning on the day of tumor implantation. Control animals received injections of the appropriate vehicle controls. Tumor volumes were measured when all animals had palpable tumors (one week post-implantation). While propranolol and DCA modestly reduced tumor growth as single agents, the combination led to a 50% reduction in tumor volume as compared to control animals at study endpoint, indicating a significant tumor growth delay (Figure 6A). Furthermore, indicative of a favorable toxicity profile, mice treated with propranolol, DCA, or the combination did not display weight loss as compared to control animals (Figure 6B). On the contrary, these animals gained a modest—but statistically significant—amount of weight versus control animals, perhaps related to the drugs’ effects on organismal metabolism. Such effects would require monitoring in the clinical setting, but are not likely to be a cause for concern as many cancer patients actually experience weight loss during the course of their disease [35,36]. In fact, weight loss is often associated with worse outcomes in cancer patients while weight stability and weight gain have been associated with improved outcomes, suggesting that therapeutic approaches that promote maintenance or gain of body weight may be beneficial [37,38]. Additionally, no overt toxicity or sickness behavior was apparent in any of the treatment groups. 

### 2.6. The Combination of Propranolol and DCA Enhances the Effects of Standard of Care therapeutic Modalities and Sensitizes Resistant Cells to Cisplatin and Radiation.

**Figure 7:**
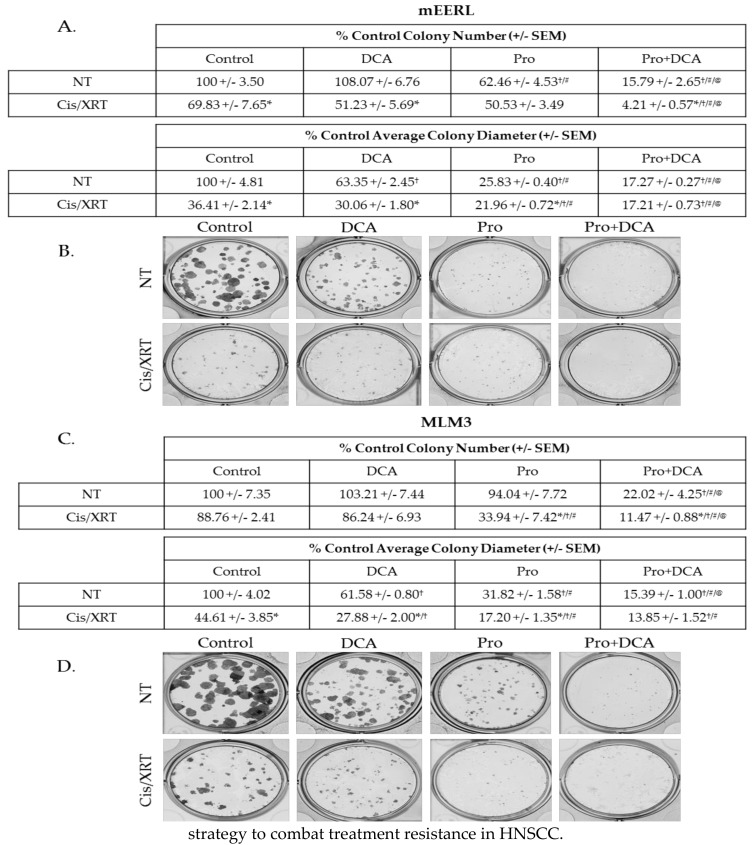
The combination of propranolol and DCA enhances the effects of standard of care therapeutic modalities and sensitizes chemoradioresistant cells to cisplatin and radiation. Colony-forming assay results and representative images of mEERL (**A**,**B**) and MLM3 cells (**C**,**D**) treated with propranolol (40 µM) and/or DCA (10 mM) concurrent with 2 µM cisplatin and 8 Gy X-ray radiation (Cis/XRT). NT = not treated with Cis/XRT. Values reflect % colony number (top) or % average colony diameter (bottom) relative to untreated controls. Each value represents mean +/- SEM of *n* = 4 independent biological replicates; *p*-values reflect *t*-test results (* *p* ≤ 0.05 comparing NT vs. Cis/XRT in the same column (e.g., DCA NT vs. DCA Cis/XRT); ^†^ *p* ≤ 0.003 vs. control cells in the same row (e.g., Control NT vs. DCA NT); ^#^ *p* ≤ 0.01 vs. DCA-treated cells in the same row; (e.g., DCA Cis/XRT vs. Pro Cis/XRT); ^@^ *p* ≤ 0.02 vs. propranolol-treated cells in the same row (e.g., Pro Cis/XRT vs. Pro/DCA Cis/XRT)).

Standard of care therapy in the clinical management of HNSCC includes a platinum-based chemotherapeutic agent, commonly cisplatin, and radiation [4]. Therapeutics that augment the effects of these modalities should improve outcomes and may allow for de-intensification of standard therapy in certain cases. To investigate the effects of propranolol and DCA alongside chemoradiation, we conducted colony forming assays treating mEERL or MLM3 cells with propranolol, DCA, or the combination with or without the combination of 2 µM cisplatin and 8 Gy X-ray radiation (Cis/XRT). While potent on its own, the combination of propranolol and DCA significantly enhanced the effects of Cis/XRT (Figure 7A–D). Even the MLM3s, which we have previously shown to be chemoradioresistant [28,32], were sensitized to the effects of Cis/XRT when treated with the combination of propranolol and DCA (Figure 7C,D). These data provide support for this novel combination as a therapeutic complement to standard cytotoxic modalities and indicate a potential strategy to combat treatment resistance in HNSCC. 

## 3. Discussion

Tumor metabolism has been recognized as a potential target for therapeutic intervention since Otto Warburg’s discovery that, even when sufficiently oxygenated, tumor cells produce copious amounts of lactate from glycolysis [39,40,41]. This finding—recognized as the “Warburg Effect”—led Warburg to conclude that dysfunctional mitochondria prevented tumor cells from utilizing more energetically efficient oxidative metabolism. Contrarily, in the many strides made since Warburg’s discovery, it is now recognized that most cancer types possess metabolically functional mitochondria whose activity is critical for tumorigenesis [42,43]. These seemingly paradoxical discoveries highlight the complexity of tumor metabolism and underscore the myriad mechanisms tumors use to meet the cellular demands for energy and biomass required to achieve sustained proliferation. The nutrient supply of the heterogeneous and ever-changing tumor microenvironment requires that tumor cells be metabolically plastic for optimal cellular fitness. Indeed, tumors exhibit diverse metabolic adaptations with significant inter- and intra-tumoral heterogeneity, including altered rates of glycolysis, glutaminolysis, fatty acid metabolism, and autophagy [21,22,23,44]. Unfortunately, while requirements for sustained proliferation in harsh conditions may render tumor cells susceptible to metabolism-targeting therapies, such plasticity and heterogeneity may significantly limit the therapeutic efficacy of single agent strategies. As such, the investigation of metabolism-targeting drug combinations is critical. 

Here, we have shown that the complementary deleterious effects of propranolol and DCA on tumor cell metabolism synergize for significant anti-cancer activity in HNSCC. We have shown that propranolol-mediated perturbations in mitochondrial metabolism sensitize tumor cells to glucose deprivation, and that tumor cell metabolism and mTOR signaling are attenuated when propranolol is combined with the glycolytic inhibitor DCA. This drug combination displays significant anti-cancer activity in vitro in both HPV(+) and HPV(−) HNSCC cell lines and significantly delays tumor growth in vivo in an aggressive R/M model of HPV(+) HNSCC. Additionally, this novel combination enhances the effects of standard therapeutic modalities and sensitizes resistant cells to cisplatin and radiation. 

Perhaps in part due to the heterogeneous nature of the disease, targeted approaches in HNSCC are sorely lacking. Cetuximab, a monoclonal antibody targeted to the epidermal growth factor receptor, remains the most clinically relevant targeted therapy in HNSCC, though its use is associated with only modest clinical benefit and response rates are poor [4,45,46]. The PI3K-Akt-mTOR axis, MAPK-ERK axis, and HIF1α are among the signaling molecules whose heightened activity in HNSCC has led to their exploration as targets for therapeutic intervention [1,4,45,47]. Notably, each of these targets are part of molecular signaling cascades that ultimately influence cellular metabolism [48]. Accordingly, HNSCC shows evidence of dysregulated metabolism [34]. Several studies have revealed metabolic signatures indicative of multiple metabolic alterations in HNSCC versus normal oral epithelium, including heightened glycolytic activity, increased anaplerotic flux, and altered lipid metabolism [49,50]. As a common point of convergence for many cancer-associated signaling processes, tumor metabolism stands as a worthy candidate for therapeutic targeting. The data presented herein supports this notion and suggests that combinatorial strategies—in this case, propranolol and DCA—are likely to be most effective. 

While the present study was limited in scope to investigating the combination of propranolol and DCA, it is likely that other agents targeting glycolysis will be similarly effective alongside propranolol. Indeed, a recent study published by Brohée, et al., found that propranolol synergized with the glycolytic inhibitor 2-DG in prostate cancer by limiting 2-DG-associated protective autophagy [51]. These data also suggest that such combinations may be effective in tumor types beyond HNSCC. Future investigations may be warranted to identify glycolytic inhibitors that most strongly synergize with propranolol without significantly worsening the toxicity profile of either drug. 

Propranolol and DCA are clinically available drugs, most notably used in the management of hypertension and lactic acidosis, respectively, and both have received interest for repurposing as oncologic agents [31,52]. As U.S. Food and Drug Administration-approved drugs, propranolol and DCA have the potential for rapid translation from the bench to the clinical oncology setting without incurring the costs associated with the development of novel therapeutics. Perhaps most importantly, both drugs are generally well-tolerated, suggesting the potential for therapeutic benefit without significantly increasing treatment-associated morbidity. These qualities, together with the data presented herein, suggest that further studies investigating the oncologic potential of this drug combination are warranted. 

## 4. Materials and Methods 

### 4.1. Cell Culture

mEERL and MLM3 cells, derived from C57Bl/6 mice, have been previously characterized [24,25,26,27,28]. SCC1 and SCC47, which were recently authenticated by short tandem repeat profiling (Genetica DNA Laboratories, Cincinnati, OH, USA), were generated at the University of Michigan (UM-SCCs) [53] and received as a generous gift from Dr. Douglas Trask (University of Iowa, Iowa, IA, USA). All cell lines were screened to ensure that they were free of mycoplasma and were maintained in Dulbecco’s modified eagle medium (DMEM; Corning, Tewksbury, MA, USA) supplemented with 10% fetal bovine serum (FBS, Atlanta Biologicals, Flowery Branch, GA, USA), 100 U/mL penicillin (HyClone, ThermoFisher Scientific, Waltham, MA, USA), and 100 μg/mL streptomycin (HyClone, ThermoFisher Scientific, Waltham, MA, USA). Primary HTE cells were obtained under an IRB approved protocol from surgically tonsillectomy of consented patients as previously described [27], and were maintained in KSFM (Gibco, ThermoFisher Scientific, Waltham, MA, USA). All cells were maintained at a humidified 37 °C in 5% CO_2_.

### 4.2. Seahorse Extracellular Flux Analysis

OCR and ECAR were measured on a Seahorse XF24 extracellular flux analyzer (Agilent, Santa Clara, CA, USA). A total of 15,000–20,000 cells/well were seeded on an XF24 microplate (Agilent) in DMEM + 10% FBS prior to overnight treatment with propranolol (40 μM, Roxane Laboratories, Inc., Columbus, OH, USA) and/or DCA (10 mM, Sigma-Aldrich, St. Louis, MO, USA). Prior to running the assay, medium was changed to pre-warmed, serum-free, sterile-filtered, pH 7.4 mitochondrial or glycolytic stress test medium (with or without propranolol and/or DCA) and cells were placed in a non-CO_2_ incubator at 37 °C for one hour. Mitochondrial stress test medium consisted of XF Medium (Agilent, Santa Clara, CA, USA) supplemented with 1 mM sodium pyruvate (ThermoFisher Scientific, Waltham, MA, USA), 2 mM glutamine (Sigma-Aldrich, St. Louis, MO, USA), and 10 mM glucose (Sigma-Aldrich, St. Louis, MO, USA). Glycolytic stress test medium consisted of XF Medium supplemented with 4 mM glutamine. XF24 sensor cartridges (Agilent, Santa Clara, CA, USA) were hydrated overnight in XF Calibrant Solution (Agilent, Santa Clara, CA, USA) at 37 °C in a non-CO_2_ incubator. Stress test reagents (Agilent, Santa Clara, CA, USA) were re-constituted in appropriate media. For the mitochondrial stress test, cells were treated with sequential injections of oligomycin (Oligo; 1 µM final), FCCP (2 µM final), and a cocktail of rotenone/antimycin A (Rot/AA; 0.5 µM final), to allow for three measurements each of basal (pre-Oligo), ATP-linked (post-Oligo), maximum (post-FCCP), and non-mitochondrial (post-Rot/AA) OCR; all values were adjusted for non-mitochondrial OCR (subtracted average of 3 measurements following Rot/AA injection) and normalized to total protein. For the glycolytic stress test, cells were treated with sequential injections of glucose (Gluc; 10 mM final), oligomycin (Oligo; 1 µM final), and 2-deoxyglucose (2-DG; 50 mM final), to allow for three measurements each of basal (post-Gluc), maximum (post-Oligo), and non-glycolytic (post-2-DG) ECAR. Instrument protocol commands were set to default. All data were normalized to total protein (quantified per well via BCA protein assay (Pierce, ThermoFisher Scientific, Waltham, MA, USA)), and are expressed as a percentage of control basal OCR or ECAR. Calculations for metabolic parameters were as follows:

Basal mitochondrial respiration is the average of the first three OCR measurements, pre-Oligo; basal glycolysis is the average of the three ECAR measurements following Gluc injection; maximum glycolytic rate is the average of three ECAR measurements following Oligo injection; glycolytic reserve is the difference between max and basal glycolysis.

### 4.3. Cellular Proliferation and Viability Assays

Equal numbers of cells were seeded in DMEM + 10% FBS in 60 mm tissue culture dishes. For glucose deprivation studies, cells were seeded in glucose-free DMEM (Gibco, #119660025; ThermoFisher Scientific, Waltham, MA, USA) + 10% FBS, (or the same DMEM supplemented with 25 mM glucose for controls), allowed several hours to adhere, and treated with propranolol (40 μM) for 48 h. For propranolol/DCA studies, cells were seeded in DMEM + 10% FBS, allowed several hours to adhere, and treated with propranolol (40 μM) and/or DCA (10 mM) for 48 h (or 72 h for the slower growing SCC47s). Culture supernatant was harvested (to collect dead/floating cells) and pooled with adherent cells harvested via trypsinization. Cells were pelleted and the resultant pellet was resuspended in DMEM in a volume appropriate to ensure cell concentration was within the accurate range of the automated hemocytometer. Cell suspension was stained at a dilution of 1:1 with 0.4% trypan blue (Gibco, ThermoFisher Scientific, Waltham, MA, USA). Live cell number and percent viability were quantified on a Countess automated hemocytometer (Invitrogen, ThermoFisher Scientific, Waltham, MA, USA). All data are expressed as a percentage of control cell number or viability for respective cell lines. 

### 4.4. Colony Forming Assays

For glucose deprivation studies, cells were seeded at a density of 200 cells/well in a 12-well plate in glucose-free DMEM + 10% FBS (supplemented with 25 mM glucose for controls). Cells were allowed several hours to adhere, prior to the addition of propranolol (40 µM) to the media for the duration of the experiment. Once all groups exhibited colonies of sufficient size, but prior to overgrowth of control colonies (4–7 days), cells were washed with PBS, fixed with 70% ethanol for 5 min, stained with crystal violet (0.5% crystal violet *w*/*v*, 10% ethanol), washed twice with PBS, and allowed to air dry. 

For propranolol/DCA studies, cells were seeded at a density of 200 cells/well in a 12-well plate in DMEM + 10% FBS. Cells were allowed several hours to adhere, prior to the addition of propranolol (40 µM) and/or DCA (10 mM) to the media. The following day, cisplatin (Calbiochem, San Diego, CA, USA), solubilized in DMSO, was added to the media to a final concentration of 2 µM (controls received an equivalent volume of DMSO vehicle). Within 15 min of cisplatin administration, cells were treated with 4 Gy X-ray radiation (RS2000 irradiator, RadSource Technologies, Inc., Buford, GA, USA). Then, 24 h following cisplatin administration and irradiation, media was replaced with fresh DMEM + 10% FBS with continued propranolol and/or DCA treatment. Once all groups exhibited colonies of sufficient size, but prior to overgrowth of control colonies (5–7 days), cells were stained as described above. Colonies were counted and measured using the GelCount system and associated software (Oxford Optronix, United Kingdom). All data are expressed as a percentage of control colony number or diameter. Control refers to 25 mM glucose/no propranolol conditions for the glucose deprivation studies, and no propranolol/no DCA/no Cis/XRT for the propranolol/DCA studies. 

### 4.5. Annexin V/Propidium Iodide Apoptosis Assay

Equal numbers of cells were seeded on 100 mm cell culture dishes in DMEM + 10% FBS and allowed several hours to adhere prior to treatment with propranolol (40 µM) and/or DCA (10 mM) for 48 h. Culture supernatant was harvested (to collect dead/floating cells) and pooled with adherent cells harvested via trypsinization. A total of 300,000 cells/sample were washed in ice cold PBS, prior to staining using FITC annexin V/propidium iodide kit (Invitrogen, ThermoFisher Scientific, Waltham, MA, USA), according to manufacturer protocol. Cells were resuspended in 100 µL annexin-binding buffer prior to the addition of FITC annexin V (3 µL/sample) and propidium iodide (0.1 µg/sample) for 15 min at room temperature. An additional 400 µL of annexin-binding buffer was added to each sample following the incubation period, to bring the total sample volume to 0.5 mL. Samples were analyzed by flow cytometry (AcurriC6, BD Biosciences, San Jose, CA, USA). Apoptotic cells were identified by annexin V positivity. Gating strategy was set using unstained samples. 

### 4.6. Western Blotting

Cells were grown to 80% confluence and harvested in lysis buffer (50 mM Tris HCl pH 7.5, 150 mM NaCl, 5 mM EDTA, 2 mN Na_3_VO_4_, 100 mM NaF, 10 mM NaPPi, 10% glycerol, 1% Triton X-100 (Tx-100)). Lysates were spun at 10,000 RPM for 10 min at 4 °C. Tx-100 soluble cell lysates were subject to BCA protein assay (Pierce, ThermoFisher Scientific, Waltham, MA, USA) and 30 μg protein/sample was separated by SDS-PAGE, transferred to a nitrocellulose membrane, incubated for 1 h in PBS + 5% bovine serum albumin, and analyzed by Western blot with antibodies against the following targets: Phosphorylated p70S6K (1:1000 in TTBS; Cell Signaling Technologies, #9206; Danvers, MA, USA); total p70S6K (1:500 in TTBS; Cell Signaling Technologies, #9202; Danvers, MA, USA); beta-actin (1:1000 in TTBS; Sigma-Aldrich, #A5316; St. Louis, MO, USA). Membrane was incubated with primary antibody overnight. HRP-conjugated secondary antibodies (1:10,000; ThermoFisher Scientific, Waltham, MA, USA) and ECL reagent (ThermoFisher Scientific, Waltham, MA, USA) were used for visualization with a CCD camera imaging system (UVP). Spot densitometry was used to quantify relative signal intensity.

### 4.7. Animal Studies

All experiments were performed in accordance with institutional and national guidelines and were approved by the Institutional Animal Care and Use Committee at Sanford Research. Briefly, 50,000 cells were injected in a volume of 100 µL DMEM subcutaneously into the right hind limb of male C57Bl/6 mice (Jackson Laboratory, Bar Harbor, ME, USA). Mice weighed 19–25 g and were ~5 weeks old. Groups were assigned arbitrarily. Propranolol (Sigma-Aldrich, St. Louis, MO, USA) and DCA (Sigma-Aldrich, St. Louis, MO, USA) were both dissolved in ddH_2_O. Mice were treated daily with 10 mg/kg/day intraperitoneal propranolol and/or 6 mg/day DCA or the appropriate vehicle (bacteriostatic 0.9% sodium chloride (Hospira, Lake Forest, IL, USA) and 2.5% ddH_2_O). Tumor volume was measured via calipers once all tumors were palpable (one week post-implantation), as previously described [54]. Animals were weighed prior to tumor implantation and again at study endpoint.

### 4.8. Statistical Analysis

SigmaPlot 14.0 (Systat Software, Inc., San Jose, CA, USA) and Excel 2016 (Microsoft, Redmond, WA, USA) were used to conduct statistical analyses. Brown-Forsythe test was used to test for equal variance (*p* > 0.05) prior to running Student’s or Welch’s *t*-test for samples with equal or unequal variance, respectively. *p*-values less than 0.05 were considered statistically significant. 

## 5. Conclusions

The results of the present study indicate that propranolol, a non-selective beta-blocker that has received interest for its anti-cancer potential, promotes glucose dependence in HNSCC and synergizes with glucose deprivation for enhanced anti-cancer activity. This phenomenon is replicated by targeting glycolytic activity with the clinically available drug, DCA, concurrent with propranolol treatment. This drug combination also enhances the effects of standard chemoradiation. Thus, this study identifies a novel, metabolism-targeting, combinatorial treatment strategy in HNSCC.
